# 

**Published:** 1891-08-01

**Authors:** 


### NOTICE TO CORRESPONDENTS.

All MS., Letters, Books for Review, and other matters intended for the Editor should be addressed THE EDITOR, THE LODGE, PORCHESTER SQUARE, LONDON, W.

The Editor cannot undertake to return rejected M.S., even when accompanied by stamped directed envelope.

**Notice to Advertisers and Subscribers.**

All Advertisements, Orders for Copies of THE HOSPITAL, and Business Communications should be addressed to The Publisher, *not to the Editor*, THE HOSPITAL, 140, Strand, W.C.

The Cost of Subscription, post free, is as follows:—Per week, 1½d.; per quarter, 1s. 8d.; per half-year, 3s. 3d.; per annum, 6s. 6d. Foreign:—Per annum, 8s. 8d.; payable, in all cases, in advance.

**NOTICE.—The Index for Vol. IX., (ending March, 1891) is on sale at the Office of this Journal, price 2½d., post free.**

### General Charities.

[This Column is devoted to an account of work of charitable institutions other than Medical Charities. The Editor will be glad to receive reports or news of work at 140, Strand. *Philanthropy* should be plainly written on the envelope.]

The Prince of Naples has sent £10 to the Hon. Secretary of the Naval Exhibition, to be devoted to the Charity connected with the Exhibition.

The season of public dinners and anniversary festivals has now practically come to a close, and that portion of the public which is inclined to good deeds may well ponder and reflect upon the results of its well-intentioned efforts. A closer study of the reports and balance-sheets of the various institutions which it has supported might be as advantageous to its pockets as it would be disadvantageous to the future of some societies. Unfortunately the readiness to take some trouble over inquiry before giving, even if present, is too easily overcome; and falls a prey to the first specious appeal. It is as easy to draw up a specious appeal as to pen a bogus prospectus, and there are too many of them abroad. It seems as though a different standard of morality holds good of the management of societies from that applied to the conduct of private affairs or business. Ugly words are used with reference to the conduct of an undischarged bankrupt who continues to trade on credit, and unpleasant but not unjust penalties occasionally attach to this method of procedure. On the other hand, societies which are notoriously in debt, in many cases through bad management in the past, or extravagant and unjustifiable expenditure, which is tantamount to the same thing, are not satisfied with appealing to the public to help them out of their debt. Of an appeal with this object no one can with justice complain. Oblivious of the fact that they are unable to meet their ordinary expenditure, far too many societies launch out into building or new schemes, relying on the generosity of a thoughtless public. This evil—and its existence is undeniable—has been creeping into the management of many otherwise well-conducted charities, and will last so long as the public will not take the trouble to look into matters thoroughly and systematically. The remedy is in their hands; will they adopt it? There are still a few charities left which have an honourable record of even a century or more, during which they have never contracted a debt beyond their means to pay. It would be well if more were to follow the example of the **British and Foreign Sailors' Society**, which has always resisted the temptation of extending its operations until it has secured the means to carry out its plans. The rule with most societies is—first extend your operations, then trust to chance and the emotional side of mankind's character in order to pay for the expenditure incurred. An auxiliary institute for seamen is to be provided by this society in Millwall, the foundation-stone of which has been laid by Lady Brassey, close to the entrance of the Millwall Docks. The inhabitants of the Isle of Dogs have contributed £100 towards the cost of the building, and Louisa Lady Ashburton the sum of £1,200.

The foundation-stone of the **Polytechnic for South-West London** was laid by the Prince of Wales on the 23rd ult. The object of the institution is to promote the intellectual skill, general knowledge, health, and well-being of young men and women in the populous district of South-West London, north of the Thames. In order to carry out this laudable end instruction will be imparted in the general rules and principles of the arts and sciences applicable to any handicraft, trade, or business, in such branches of art, science, language, literature, and general knowledge as shall be approved by the governing body, and of such a kind as will prove of use to those who intend to emigrate. Facilities will be granted for various forms of muscular exercise, such as swimming and gymnastics, and the formation of clubs and societies, and there will be a library, museum, and reading-rooms. The most important, and not the least useful, feature will be the formation of day schools and evening classes for boys and girls between the ages of fifteen and thirteen, in commercial, scientific, and general education. Three years ago the needs of the district and its claims for a grant from the London City parochial funds were urged upon the Charity Commissioners. The favourable reception of the scheme by this body was followed up by the gift of a site in the Manresa Road, a central part of the district, near the Chelsea Free Library, by Lord Cadogan, the value of which is £10,000. The Charity Commissioners then promised a sum of £50,000 towards the endowment and support of the institution, on condition that the public should give a similar sum for the site, building, and equipment. Towards this the sum of £34,120 has, up to the present time, been raised. His Royal Highness, who re-echoed the hope of the Committee that the remaining £16,000 would soon be subscribed, pointed out that although in so wealthy a district, which includes the parishes of Fulham, Chelsea, St. George's, Hanover Square, Kensington, and Westminster, no great difficulty should be found in obtaining the sum, it is not so easy a matter as some would suppose. In the first place, few people realise how much poverty exists in this part of London, and secondly, appeals on behalf of philanthropic schemes are constantly being made to the wealthy inhabitants of the district from all quarters of the metropolis and the country at large, so that they are frequently disinclined to take any special interest in claims for aid from their own locality.

### Scraps and Gleanings.

Miss Talbot, of Morgam Abbey, is about to build a cottage hospital at Port Talbot, South Wales.

The Harrow Cottage Hospital continues to prosper. The paying ward and provident fund are much appreciated.

It has been decided to erect a Cottage Hospital at Galashiels, towards which fourteen gentlemen have contributed £1,700.

Affairs are not yet quite settled at the Ashford Cottage Hospital, and dissatisfaction seems still rife amongst the medical staff.

At the Dunoon Convalescent Homes 3,338 patients have been received during the past year. The expenditure has amounted to £6,318.

At the seventy-fourth annual meeting of the Swansea Hospital it was resolved to organise a special department for the diseases of women.

Blackpool is becoming alive to the fact that it much needs a hospital At present serious accident cases are removed twenty miles to Preston

Over £5,000 has already been promised towards the proposed convalescent home in connection with the Birmingham Hospital Saturday Fund.

The International Congress of Hygiene and Demography will open on the 10th of August next, under the presidency of H.R.H. the Prince of Wales.

The Warwickshire County Council and the Rugby Local Government Board are carrying on a guarded fencing match on the subject of the proposed Epidemic Hospital for Rugby.

Sir Henry Roscoe reports favourably upon a series of experiments he has made of depriving the air entering the Houses of Parliament of its living germs, by passing it through cotton wool.

At the last quarterly meeting of the Bedford General Hospital it was announced that the Duke of Bedford had consented to become a House Visitor at the hospital, and that the Matron, Miss Newberry, had resigned.

The Police Commissioners of Dunfermline have resolved to provide a contagious diseases hospital for the burgh, and the District Committee of the County Council have arranged for the erection of a temporary building.

The Police Commissioners of Dunfermline have resolved to provide a contagious diseases hospital for the burgh, and the district committee of the County Council have arranged for the erection of a temporary building.

The Stockton Hospital Building Committee have decided not to add to the contracted sum for the building of the new wing of the Hospital, on which Mr. Laing, the contractor, is stated to have lost between £800 and £400.

The Committee of the Leicester Infirmary have recognised the substantial benefits conferred on that institution by the Sports Committee of the town, by electing two of its members Life Governors of the infirmary.

The Birmingham and Midland Eye Hospital is doing good work, and further accommodation is needed. The number of subscribers continues to increase steadily, and the accounts show a surplus over the expenditure.

The report for 1890 of the Grosvenor Hospital for Women and Children shows that the work of the hospital is well maintained. The graduated scale of patients' payments in use at this institution is a judicious and practical one.

The Gravesend Hospital presents a favourable report for this year. The hospital continues deservedly popular, the nursing institute is making good progress, and the accounts of the Samaritan Society, under a committee of ladies, are most satisfactory.

### THE STUDENT OF MEDICINE.

[All communications for this Supplement should be addressed to The Editor of The Hospital, at 140, Strand, with the words, "Students' Column," written in the left hand top corner of the envelope.]

### ON GERMAN STUDENTS (*continued*).

*The substance of a paper read before the St. Mary's Hospital Medical Society by* W. Overend, *B.A. Oxon.*

Coming to *Pathology* the teaching is thoroughly good, and the manner of conducting a post-mortem is, like everything else in Germany, nothing if not strictly and absolutely methodical. It reminds one of the monotony of military precision when Recklinghausen (of Strasburg) conducts a post-mortem; then two assistants are present, one being his own assistant, and the other the assistant of the surgeon or physician to whom the case belonged. These sit at benches and take down the results verbatim which Recklinghausen dictates at some length as he proceeds, while the class stands round the theatre, smoking being permitted and quite the rule. The hospital physician or surgeon is also present.

Most of the fluids are measured, and the organs weighed "seriatim." Recklinghausen makes 500 or 600 post-mortems yearly, there being no difficulty about obtaining a post-mortem on any case, since the law permits it. With regard to histology Recklinghausen thinks that sections should not necessarily be limited to one plane, so that sections for the microscope, *a la* Recklinghausen, are not too thin.

Arnold, of Heidelberg, pursues a similar method (although his histological specimens were magnificent). On two afternoons during the week he demonstrated the pathological specimens which had been obtained in the interval. Having described a specimen on the board, he then took it round among the students, stopping at batches of about eight men and pointing out the salient features, microscopical specimens being passed round the whole time. About ten specimens were described in the course of each lecture. As an instance, one afternoon, he described the following: 1, Enchondroma of tibia, parts of which were ossified, forming an osteoma. 2, Fibroma of mammary gland. 3, Croupous pneumonia, with microscopic specimen of Fraenkel's diplococcus. 4, Granular kidney with formation of cysts. 5, Sarcoma of ovary. 6, Case of acute phosphorous poisoning, fatty degeneration of liver, kidney and lungs. 7, Primary cancer of gall duct. 8, Carcinoma of rectum. 9, Pachymeningitis hæmorrhagica of the dura mater. 10, Epithelioma of lower lip. All of these being accompanied by microscopic specimens. I may here mention that Arnold is especially good in pathological histology.

After the pathology lectures followed the ordinary lectures on medicine and surgery, which formed the concluding lectures of the day, and all finished about a quarter to seven p.m. The student, having finished his day's work, goes to "abendessen," or supper. Unless he is in for an examination, he is content to spend the rest of the day playing billiards, smoking, and drinking beer. Two evenings weekly will be spent with his club or corps, the principal features of which consist of singing students' songs in chorus, making of speeches, and drinking beer *ad lib.*, according to the refinements of German bacchanalian etiquette. The remaining evenings of the week will be devoted in winter to the cafés or theatres, and in summer to the beer gardens. Sunday afternoons are spent generally at a military concert or in drinking beer in some village "pub." outside the town. In Heidelberg a very favourite resort was Neckarsteinach—a small village about six miles up the river. Of course, after hearing lectures from "morn till dewy eve," for the rest of the day his capacity for imbibing knowledge is simply nil; in fact, they are lectured to death. English brains and muscles would rebel.

The students seem to realise the responsibilities of life very early, judging from their behaviour in lectures and by the interest they exhibit when discussing medical questions among themselves. A German student, however, sees very little of actual medical practice. Of course, he attends classes in bandaging, auscultation, &c., within the wards, but the functions of clinical clerk and dresser (so-called amanuensis) are optional. Moreover, these when obtained only last for a month. Take, for instance, surgery. Although the clinical lectures are excellent, and English students would rapidly benefit from them, still it is not every student that can be elected an amanuensis even if he wished. The office of these amanuenses is to give chloroform, help a little in the operation and bandaging. The giving of chloroform by the amanuensis is somewhat casual. On more than one occasion I have seen Czerny obliged to stop in the middle of the operation

### THE STUDENT OF MEDICINE—(*continued*).

owing to spasm of the respiratory muscles, &c., and to seize the opportunity of giving a word of advice as well as rebuke.

Czerny also calls the men in turn (so-called Practikanten) down into the area of the theatre to examine cases before him, but very few seem to know anything about the subject. Czerny takes the men once a week for about half an hour around the wards, very few of the men come, as they do not seem to appreciate it as we do. Although these clinical lectures are excellent in their way, since they combine theory with practice, still they are utterly unable to compensate for the daily observation of patients in the wards which our own clerks and dressers must make. It follows, therefore, that the German students after their final examination, although they possess an excellent acquaintance with the theory of medicine, possess little actual experience. Of course, if what our older practitioners tell us is true, namely that they knew nothing when they passed their final, but have learnt everything since they became responsible individuals, then the German student is not much to be commiserated. They certainly possess a scientific basis to work upon, and are up to all the latest wrinkles as regards therapeutics, forgetting the oldest reliable drugs, so much so that Erb lost his patience on more than one occasion.

Our own students when they leave the hospital are practical men, well equipped for their requirements; our examinations are severe and stringent, and such of our students as complete a house appointment can compare favourably with our older practitioners, though they do not, perhaps, possess the tact of dealing successfully with functional disorders and fretful patients.

### NOTES FROM THE SCHOOLS.

#### University of Aberdeen.

The candidates for the vacant chair of Medicine at this University are now Dr. Angus Fraser, Aberdeen; Dr. Alex. Macgregor, Aberdeen; Dr. Blaikie Smith, Aberdeen; Dr. Middleton, Glasgow; and Dr. Finlay, London. It is probable that the appointment will be made before Parliament rises. End of the term. The summer session of the medical school closed on July 10th, and the examinations began the following day, and will be continued for a fortnight.

### APPOINTMENTS.

At Guy's Hospital, R. S. Freeland, M.R.C.S., L.R.C.P., and H. B. Rygate, M.R.C.S., L.R.C.L.P., have been appointed house surgeons; H. Hodgson, M.R.C.S., L.R.C.P., and W. G. Rogers, M.B.Lond., assistant house surgeons; and J. S. Richards, M.B., B.S.Lond., and W. Winslow, M.B., B.C.Camb., have been appointed house physicians. At the Victoria Hospital for Children, H. D. Rolleston, M.A., M.B. Camb., M.R.C.P.Lond., has been appointed physician. At the Westminster Hospital, H. W. Gordon Green, M.R.C.S., L.R.C.P., has been appointed senior house surgeon.

### VACANCIES.

The following vacancies are announced this week, the amount of salary in each case being given after the name of the institution:

#### Resident Medical Officers.

City Asylum, Birmingham, resident clinical assistant—Board, &c.
City of London Hospital for Diseases of the Chest, Victoria Park, E., House Physician—Board, &c.
Devon County Lunatic Asylum, Assistant Medical Officer—Salary £120 and board, &c.
Devon and Exeter Hospital, Two Resident Pupils.
General Hospital, Birmingham, three House Surgeons—Board, &c.
Hospital for Consumption and Diseases of the Chest, Brompton—House Physicians.
Metropolitan Asylums Board, South Eastern Fever Hospital, New Cross Road, Clinical Assistant for three months—Board, &c.
Newport and County Infirmary and Dispensary, Newport, House Surgeon—Salary £100 and board, &c.
Nottingham Borough Asylum, *locum tenens* for a month, from the middle of August—Salary 2 guineas a week.
Royal Albert Edward Infirmary and Dispensary, Wigan, Senior House Surgeon—Salary £100 and board, &c.
St. Luke's Hospital for Lunatics, Old Street, two Clinical Assistants—Board, &c.
Salisbury Infirmary, House Surgeon—Salary £100 and board, &c.
Wolverhampton and Staffordshire General Hospital, Wolverhampton, House Surgeon, also House Physician—Salaries £100.

#### Non-Resident Medical Officers.

Abbey Parochial Board, Paisley, Medical Officer for the Eastern District—Salary £35 with fees.
Applecross Parochial Board, Medical Officer—Salary £95.
Chelsea Hospital for Women, Fulham Road, Anæsthetist.

### THE STUDENT OF MEDICINE—(*continued*).

Clergy Mutual Assurance Society, Physican.

Hampstead Provident Dispensary, New End, Medical Officer.

London Throat Hospital, Great Portland Street, two Clinical Assistants for three months.

Manchester Royal Infirmary, Junior Administrator of Anæsthetics—Salary £50.

Queen's College, Birmingham, Lecturer on Operative Surgery.

Wolverhampton and Staffordshire General Hospital, Wolverhampton, Honorary Surgeon.

### SCHOLARSHIPS AND PRIZES.

**Royal College of Science, London.**

The following scholarships and prizes have just been awarded: First Year's Scholarships—William Allan, Thomas T. Bedford, Edwin Edser, and Herbert A. Clark. Second Year's Scholarships—John W. Pickles and Sydney Whalley. Edward Forbes Medal for Biology—Arthur G. Butler. Murchison Medal for Geology—Charles G. Cullis. Tyndall Prize for Physics, Course I.—William Allan. De la Beche Medal for Mining—James G. Lawn. Bessemer Medal for Metallurgy—Joseph Jefferson. Frank Hatton Prizes for Chemistry—Herbert Grime and Lionel M. Jones. Prizes given by the Department of Science and Art: Mechanics—Charles H. Kilby. Astronomical Physics—Charles P. Butler, Herbert A. Clark, Lawrence Parry, and Samuel S. Richardson. Practical Chemistry—William A. C. Rogers. Mining—James G. Lawn. Principles of Agriculture and Agricultural Chemistry—Henry Wilkinson.

### PASS LISTS.

**Royal Colleges of Physicians and Surgeons, Glasgow.**

The quarierly examinations in connection with the Royal Colleges of Physicians and Surgeons of Edinburgh and the Faculty of Physicians and Surgeons, Glasgow, took place this month with the following results:—

First Examination.—Of 54 candidates the following 43 passed: Alfred Vernon Crompton Holt, Cheshire; Thomas Owen Jones, Anglesea; Henry Frederick John Graves, Birmingham; Michael Ryan, Dripsey; Kate Isabel Clutterbuck, Somerset; Ella Stewart Caldwell, Edinburgh; Elizabeth Taylor Gilchrist, Greenock; John Samuel Sutton Maher, County Tipperary; Mary Ross M'Dougall, Strathpeffer; Elizabeth Marianne Erskine, Glasgow; Lillias Jane Thomson, Montrose; Charles Sumner Edwards, Staffordshire; Amy Gordon Lillingston, Australia; John Forrest Sutcliffe, County Kildare; Edwin Charles Hayes, Dublin; Margaret Ferooza Macnaughton, Edinburgh; Isabella Beattie Venters, Blairgowrie; David Alexander, Jamaica; William Heatlie Ferrier, Inveresk; Arthur O'Leary, County Cork; Mary Buchanan Lee, Liverpool; Robert Bowles Sandiford, Durus, County Cork; Minnie Ethel Bowlby, Sandhurst; Robert Strickland Hannay Fuhr, Belfast; Martha Florence Armitage, Staffordshire; Beatrice Ritchie, Perth; Hugh Gillies, Argyllshire; Holland May Harrison, Liverpool; William Henry Griffith, Flintshire; Beatrice Garvie, Perth; David Dickson Muir, Edinburgh; John Pritchard, Bangor; Cyril Purdon, Belfast; William Edward Vice, South Africa; Walter Ernest Duckworth, India; Walter Kirkby, Lincolnshire, Henrietta Kate Cornford, Gloucestershire; Matthew Wilson, Linlithgowshire; Richard Dagger, Lancashire; Albert Harding Rand Porter, New Zealand; Lewis Tyrer Jones, Anglesey; David Charles Rowlands, Swansea; and John James Healy, Victoria.

Second Examination.—Of 53 candidates the following 30 passed: Patrick M'Elwaine, Cavan; Herbert Hewlett Isherwood, Preston; Norman Gladstone Douglas, Crewe; James Louis Scott Sherlock, Worcestershire; Samuel Percival Smith, Swindon; George Batchin Thompson, Edinburgh; James Kitchin, Elgin; Marsham à Beckett M'Carthy, Sydney; Daniel John Backley, Cork; John Noonan, Cork; Elias Jeffery, Falmouth; Wilfrid Lawson, Hull; James Henry Dewhurst Stephenson, Blackburn; William Dill, Cork; James Francis Clark Hossack, London; Catherine Mabel Blackwood, Edinburgh; Robert Fair, County Galway; William Yeates, County Down; Herbert Arthur Howes, Sale, Cheshire; John Larwill, Sussex; Patrick Sullivan, Cork; Michael Ryan, Cork; John Besnard Wilson, Cork; Thomas Francis Roche, Cork; Walter Somerville, Cumberland; James Martin, Lanarkshire; Louisa Charlotte Nash, Bombay; Ernest Switzer Forde, Cork; George Fortescue Jackson, Kingstown; and John Murley Rendall, Torquay.

Final Examination.—Of 84 candidates, the following 43 passed, and were admitted L.R.C.P. and S.E., and L.F.P. and S.G.: Joshua Hamilton Hart, Yorkshire; Mark Ringwood Rich, Clonmel; Daniel Albert Rose, Montreal; Herbert Danvers, Cape Town; Arthur Thomas Kember, South India; Ainslie Power Ardagh, Canada; Edward Edwards, Anglesea; Charles Frederick Weeks, Hull; Alexander Henry Dudley Salt, India; William Charles Ellis, Stoke, Devon; Nevil Pottow Cadel, Faringdon; David Kidd Muir, West Hartlepool; Alfred Jackson, County Cork; James Fox, County Tyrone; Emily Charlotte Thomson, India; Herbert Arthur Howes, Sale, Cheshire; Frederick William Marsden, Russia; Jane Marsh, Hilton, Dorset; Charles Alexander Macnab, Lanarkshire; Elizabeth Christie, Glasgow; Thomas Bernard Kelly, Galway; James Downe Ross Watt, Leamington Spa; Henry Robert Brown, Bangalore; Ernest Coleman Moore, Bristol; George Bidie, Fochaber; Henry Chestnut, Tralee; Alice Margaret Moorhead, Maidstone; Florence Gertrude Wells, Hosur; Henry Arthur Giles Hadden, Wexford; Sydney Henry Merryweather, Yorkshire; John David Hadden, India; Edwin Dacre Dunn, Redcar, Yorkshire; Ernest George Salt, Norfolk; William Fingland, Liverpool; Edward William Sharman, Rutland; Paget Dundas Minchin, Kilkenny; Timothy White, Ballymena; Samuel Moore Giffen, Belfast; Joseph Gordon-Smith, St. Andrews, Jamaica; William Forsaith Bauchop, New Zealand; John Cuthbertson Walker, Helensburgh; John Henry Boland, Dundee; and Edward Hughes, Canarvon.

### THE STUDENT OF MEDICINE—(*continued*).

### Royal Colleges of Physicians and Surgeons of England.

The following gentlemen passed the second examination of the Board in anatomy and physiology on the 15th ult.: Messrs. Sidney Hughes, Alfred Miller, John Claude Verity Wilkins, and Joseph Clarence Stanhope Peaton, Guy's Hospital; William Jenkin Evans, Robert Kerr Hamilton, Louis Edward Dartnell, Charles Albert Jones, Richard Jones, James Henry Jolley, Frederick Charles Simpson, and Henry Russell Andrews, London Hospital; Frederick William Kerbey, London Hospital and Mr. Cooke's School of Anatomy and Physiology; Albert Otto Bobardt, Melbourne University; Henry Hugh Powell Cotton and Reginald Nitch-Smith, Westminster Hospital; Charles Henry Russell, Charing Cross Hospital; Gerald Robert Badwin and Christopher Dering Dawes, St. George's Hospital; Cecil Butler Simpson, St. Thomas's Hospital; Archibald Hugh Payan Dawney and Charles Henry Dissent, University College; Clement Arthur Newbold, St. Bartholomew's Hospital; and Robert Henry Hogg, Otago University.

The following gentlemen passed in Anatomy only: Messrs. James Evan Jones and Archibald Walter Lamb, St. Bartholomew's Hospital; Frederick Victor Elkington, St. Bartholomew's Hospital and Mr. Cooke's School of Anatomy and Physiology; Francis Joseph Loveday and Rees Gabe Jones, London Hospital; William Drake and Frederick Henry Elworthy Anning, University College; Walter Llewellyn Roberts, St. Mary's Hospital; and Hubert Hospe Thomas, Walter Harmon Morgan, and William Robert Larbalestier, Charing Cross Hospital.

The following gentlemen passed in Physiology only: Messrs. Edward Gurdon Frederick, King's College; Charles William Lanphier, William Escombe, Arthur Godfrey Ince, and Matthew Carter, Charing Cross Hospital; Gwilym Lewis, Joseph Edgar Sydney Old, Francis Edward Bromley, Philip George Williams, and Dan Davies, London Hospital; Walter Lea Stuart, Guy's Hospital; John Hay Campbell and John Burnett Yelf, St. Mary's Hospital; Arthur Norris Wilkinson, Westminster Hospital; Leonard Harman, St. Thomas's Hospital; and Walter Mawer, St. Bartholomew's Hospital.

Passed on the 16th inst.:—

Anatomy and Physiology.—Joseph C. S. Peetson, of Guy's Hospital; Cecil B. Simpson, of St. Thomas's Hospital; Frederick C. Simpson and Henry R. Andrews, of London Hospital; Frederick W. Kerby, of London Hospital and Mr. Cooke's School of Anatomy and Physiology; Albert O. Bobardt, of Melbourne University; Charles H. Russell, of Charing-cross Hospital; Clement A. Newbald, of St. Bartholomew's Hospital; Robert H. Ogg, of Otago University.

Anatomy Only.—Francis J. Loveday, of London Hospital: Frederic V. Elkington, of St. Bartholomew's Hospital and Mr. Cooke's School of Anatomy and Physiology; Walter L. Roberts, of St. Mary's Hospital; Walter H. Morgan and William R. Larbalestier, of Charing-cross Hospital.

Physiology Only.—Edward G. Frederick, of King's College; Charles W. Lanphier, William Escombe, and Arthur Godfrey Ince, of Charing-cross Hospital: Francis E. Bromley, Phillip G. Williams, and Dan Davies, of London Hospital; Walter L. Stuart, of Guy's Hospital; John H. Campbell and John B. Yelf, of St. Mary's Hospital; Arthur M. Wilkinson, of Westminster Hospital; Leonard Harman, of St. Thomas's Hospital.

### Society of Apothecaries of London.

The following candidates have passed in Surgery: C. C. S. Barry, St. George's Hospital; E. H. Bingley, St. Mary's Hospital; F. Dove, London Hospital; W. F. L. Green, King's College Hospital; A. E. E. Jones, Middlesex Hospital; J. Kyffin and M. C. Langford, London Hospital; F. W. Pogson, Leeds, Yorkshire College; E. G. Renny, St. Thomas's Hospital; A. Richardson, University of Edinburgh; P. J. Ryan, University College; D. Sims, St. Thomas's Hospital; C. C. Vigurs, Cambridge University and St. Mary's Hospital; G. C. W. Williams and E. H. Willock, St. Thomas's Hospital.

The following have passed in Medicine, Forensic Medicine, and Midwifery: W. Anderson, University of California; A. C. Dornford, London Hospital; J. George, King's College Hospital; W. C. Hinde, Middlesex Hospital; E. J. Steegmann, St. Mary's Hospital.

In Medicine and Midwifery.—E. H. Bingley, St. Mary's Hospital; R. A. Earle, Middlesex Hospital; T. H. English, London Hospital; H. J. Forster, Westminster Hospital; A. F. Gervis, St. Thomas's Hospital; T. W. Smith, King's College Hospital; C. F. Warren, St. Mary's Hospital.

In Forensic Medicine and Midwifery.—A. G. Haydon, St. Bartholomew's Hospital; A. W. C. Herbert, St. Mary's Hospital; S. Melville, University College.

In Medicine.—H. C. Coopland, St. Bartholomew's Hospital; C. O'Sullivan, London Hospital.

In Forensic Medicine.—R. Jackson, London Hospital; G. C. W. Williams, St. Thomas's Hospital; E. H. Willock, St. Thomas's Hospital.

In Midwifery.—S. A. E. Griffiths, Middlesex Hospital; G. F. Knipe, Liverpool University College; P. G. Laver, St. Thomas's Hospital.

To Messrs. Anderson, Coopland, Dornford, George, Jackson, Knipe, Melville, Richardson, Ryan, Williams, and Willock was granted the diploma of the society entitling them to practise medicine, surgery, and midwifery.

### Royal Colleges of Physicians and Surgeons, Ireland: Conjoint Scheme.

The following have passed the Second Professional Examination: G. H. Ayres, J. Behane, J. G. Burne, Moore Betty, J. F. A. Bulfin, J. Campbell, W. Ceneys, J. J. Costello, T. E. Cottu, W. S. Crosthwait, M. J. Cuffe, J. J. Dolan, C. P. Haunen, R. J. Harvey, G. J. Houghton, R. D. Jephson, P. King Joyce, F. J. Matthews, H. H. Moffatt, Denis Molyneux, J. R. O'Brien, J. H. Power, W. F. Roe, Charles Skelly, R. Somerville, Hubert R. Sweeny, W. Taylor, Elizabeth A. Tennant, A. R. Twigg, H. G. Thomson, and J. R. C. Whitley.

